# Effects of beta and gamma radiation sterilization on growth factor-loaded nanoparticles: an innovative approach for osteoarticular disorders treatment

**DOI:** 10.1007/s13346-025-01829-5

**Published:** 2025-03-11

**Authors:** Jorge Ordoyo-Pascual, Sandra Ruiz-Alonso, Idoia Gallego, Laura Saenz-del-Burgo, Jose Luis Pedraz

**Affiliations:** 1 NanoBioCel Research Group, Laboratory of Pharmacy and Pharmaceutical Technology, Department of Pharmacy and Food Science, Faculty of Pharmacy, University of the Basque Country (UPV/EHU), Paseo de la Universidad 7, Vitoria-Gasteiz, 01006 Spain; 2 Bioaraba, NanoBioCel Research Group, Vitoria-Gasteiz, 01009 Spain; 3 Networking Research Centre of Bioengineering, Biomaterials and Nanomedicine (CIBER-BBN), Institute of Health Carlos III, Madrid, 28029 Spain

**Keywords:** Osteoarticular disease, Nanoparticles, Growth factors, Release profile, Vascularization, Cell proliferation

## Abstract

The prevalence of various diseases, including osteoarticular conditions, is increasing as the world’s population ages. These disorders lead to degeneration of bones and joints, diminishing the quality of life of the geriatric population and imposing a significant economic burden on healthcare systems. The aim of the present study is to sterilize nanostructured lipid carriers (NLCs) loaded with vascular endothelial growth factor 165 (VEGF165) and platelet-derived growth factorBB (PDGF-BB) without compromising their properties to improve osteoarticular disease prognosis. Therefore, two methods of sterilization using ionizing radiation - beta radiation and gamma radiation - and two different doses – 12 kGy and 25 kGy - were investigated. Subsequently, the study evaluated whether the sterilization process had any effect on the nanoparticles and encapsulated growth factors by assessing their physicochemical properties, toxicity, release profiles and bioactivity. The treatment with 12 kGy of beta radiation successfully sterilized the batch of nanoparticles without inducing any changes in the physicochemical properties. In addition, the release profile of VEGF165 remained unchanged, although a slight decrease was observed in the case of PDGF-BB. The biological activity of the growth factors showed a slight decrease, with the most effective concentrations being 5 ng/mL for VEGF165 and 50 ng/mL for PDGF-BB. Taken together, these findings suggest that the nanoparticles loaded with VEGF165 and PDGF-BB can be successfully sterilized while retaining both their properties and biological activity. These nanoparticles may offer a promising new approach for the treatment of osteoarticular diseases by enhancing vascularization and promoting cellular proliferation in the affected tissue.

**Graphical Abstract:**

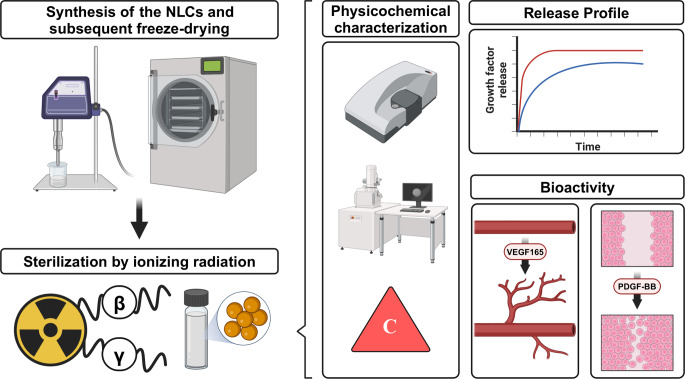

**Supplementary Information:**

The online version contains supplementary material available at 10.1007/s13346-025-01829-5.

## Introduction

Osteoarticular diseases constitute a type of degenerative bone disease that affects both the structure and function of bones and joints. The metabolic activity of bone tissue declines with age, disrupting the balance of bone metabolism and leading to disorders of bone and joint function [1]. Among these diseases, the most prominent are osteoarthritis (OA) and rheumatoid arthritis (RA) [2]. The prevalence of these diseases is increasing every year as the world’s elderly population grows [3], and it is expected that they will become one of the major contributors to the global morbidity burden in the future [4]. In addition, the incidence of these diseases in younger age groups has increased in recent years [5]. The economic burden on healthcare systems is important, with estimated annual costs per patient amounting to $5,712 for OA and $9,348 for RA [6]. Current treatment of these conditions primarily focuses on symptom management and attempts to slow disease progression, highlighting the urgent need for new therapeutic approaches [7, 8]. However, recent advances suggest that cartilage regeneration through tissue engineering techniques may offer a potential treatment for osteoarticular diseases [9]. The development of 3D bioprinting has enabled the integration of cells and growth factors into bioprinted scaffolds, thereby enhancing therapeutic options. Specifically, the use of growth factors such as Vascular Endothelial Growth Factor 165 (VEGF165), which promotes cellular angiogenesis and Platelet-Derived Growth FactorBB (PDGF-BB), which stimulates cellular proliferation, may improve the prognosis of osteoarticular disease.

Growth factors, identified as cytokines with the ability to induce cell proliferation, migration, differentiation, growth and activation, exert their influence by binding to specific receptors located on the cell membrane. These interactions activate various intracellular signaling pathways that culminate in the increase of protein synthesis processes and consequently, the generation of different biological responses [10]. However, it is important to consider the inherent instability of growth factors, which makes them susceptible to rapid degradation by enzymes, temperature changes, pH fluctuations and reactive oxygen species (ROS) [11]. As a result, achieving the required therapeutic effects may require the use of higher concentrations or prolonged exposure times, increasing the potential for adverse side effects [12].

To reduce this instability, encapsulation of these growth factors within nanoparticles (NPs) offers a protective measure. NPs are specifically defined as particles characterized by dimensions on the nanometer scale [13]. In the field of biomedicine, they have versatile applications in the diagnosis, treatment and prevention of diseases. In particular, NPs are currently used in the medical field and play a key role in gene and drug delivery, pathogen and protein detection and the advancement of tissue engineering, among others [14]. Variations in the composition of their synthesis lead to the formation of different types of NPs, each characterized by specific properties. Among these, nanostructured lipid carriers (NLCs) exhibit a number of notable advantages. In particular, they have the ability to encapsulate both hydrophilic and hydrophobic compounds, have a significant drug loading capacity and offer the possibility of modulating the release kinetics of the encapsulated substance. In addition, NLCs offer extended shelf life and are well suited for efficient large-scale industrial production [15]. Moreover, the encapsulation of growth factors within these NPs serves as an effective protective measure against degradation by external agents [16].

Currently, much research focuses on nanoparticle encapsulation of growth factors for clinical benefit [17–20]. However, there is a lack of research into the sterilization of these nanoparticles, which represents a significant barrier to future studies, as it is a critical requirement for clinical translation. To ensure that NPs are sterile, it is essential that they are subjected to a sterilization process capable of eliminating all microorganisms, including spores [21]. The most commonly used sterilization methods are sterile filtration, autoclaving, non-ionizing radiation (such as infrared and ultraviolet radiation) and ionizing radiation (such as gamma and beta radiation) [22]. The use of gamma and beta radiation is considered to be one of the most efficient techniques for reducing microorganisms without causing a drastic increase in temperature [23]. Although it is one of the most commonly used methods, there is still a risk of damaging the sample, particularly if it is unstable [22]. This damage may be induced by the generation of ROS that arise when the sample is exposed to this type of radiation [24]. Consequently, the stability of the chemical bonds within biological macromolecules, including lipids, proteins, and nucleic acids, may be adversely affected [25]. Moreover, the stability of active compounds, such as VEGF165 and PDGF-BB, may also be compromised, with higher doses leading to increased damage to the growth factors [26, 27]. The application of these radiations may induce alterations in the physicochemical and biological properties of NLCs, thereby impacting their efficacy. Consequently, it is important to investigate the effects of both radiation types on the nanoparticles as well as the encapsulated factors.

In addition to their applications in the treatment of osteoarticular diseases, NLCs loaded with growth factors exhibit significant potential in the emerging fields of tissue engineering and 3D bioprinting [28, 29]. 3D bioprinting is considered a promising method for producing biomimetic scaffolds that are important for facilitating cell proliferation and differentiation. These scaffolds have the intricate 3D architectures required for a range of tissue engineering applications [30]. The incorporation of NLCs into scaffolds could provide controlled and localized release of growth factors, thereby enhancing the regeneration of complex tissues and the integration of grafts into 3D models.

The first objective of this study was to sterilize empty nanoparticles without compromising their stability. To achieve this, empty NLCs were analyzed before and after irradiation with beta and gamma radiation at doses of 12 kGy and 25 kGy by studying changes in their physicochemical properties. The study then investigated the impact of radiation on the encapsulated growth factors, both VEGF165 and PDGF-BB. Changes in their release from the nanoparticles were investigated and any effect on their intrinsic bioactivity after sterilization was assessed. Finally, NLCs loaded with VEGF165 and PDGF-BB were successfully obtained, maintaining both sterility and biological activity. The combined action of these growth factors promotes vascularization and cell proliferation in the affected area, presenting a promising treatment option for patients with osteoarticular disease.

## Materials and methods

### Preparation of nanostructured lipid carriers

NLCs were prepared employing the emulsification-ultrasonication method, involving two phases: a lipid phase with 10 mg of Miglyol 812 N (IOI Oleochemicals, Hamburg, Germany), 100 mg of Precirol ATO 5 (Gattefossé SAS, Madrid, Spain) and 100 µg of VEGF165 or PDGF-BB (STEMCELL Technologies, Vancouver, Canada); and an aqueous phase with 20 mg of 1.3% (w/v) Tween^®^ 80 (Panreac Chemicals, Barcelona, Spain) and 10 mg of 0.6% Poloxamer 188 (BASF, Ludwigshafen, Germany) dissolved in 1.5 mL of MilliQ water. The lipid phase was melted in a 65 ºC water bath, combined with the aqueous phase, and sonicated for 30 s. After gradual cooling and overnight incubation at 4 ºC, the NPs underwent a washing procedure to isolate any unencapsulated factor using amicons (Sigma-Aldrich, Saint Louis, USA). The liquid fraction was collected for encapsulation efficiency quantification (EE). This process was repeated three times. The NLCs were then transferred to a glass vial and a 15% (w/w) trehalose solution (Sigma-Aldrich, Saint Louis, USA) was added as a cryoprotectant, followed by freeze-drying (Telstar Lyobeta freeze-dryer, Terrassa, Spain). For the freeze-drying process, the chamber was first frozen to -50 ºC over a period of 3 h. Subsequently, a pressure of 0.2 mbar was applied for 5 h. This was followed by the primary drying step where the temperature was gradually increased from − 50 ºC to 20 ºC over a period of 7 h. Finally, the secondary drying continued with the temperature maintained at 20 ºC and the chamber depressurized, and the samples were kept under these conditions for 24 h.

### Physicochemical characterization of NLCs

Nanoparticle characterization was performed using the Zetasizer Nano ZS (Malvern Instrument, UK). Dynamic light scattering determined particle size and polydispersity index (PDI), while Laser Doppler Velocimetry quantified zeta potential. To achieve this, 1 mg of NLCs were resuspended in 100 µl of MilliQ water and subjected to vortex mixing, followed by 5 min of ultrasonication in a water bath. The process was repeated and an additional 10 min of ultrasonic treatment were applied. Finally, 10 µl of the sample were diluted to 100 µl with Milli-Q water and the resulting sample was carefully placed in a measurement cell and introduced to the Zetasizer equipment.

The analysis of nanoparticle morphology was conducted using cryo-transmission electron microscopy (cryo-TEM) within a Talos F200i (FEI) instrument, operating at an accelerating voltage of 200 KeV, employing both brightfield and low-dose image modes. A 3 µl aliquot of the sample solution was applied to 300 mesh Quantifoil TEM grids that had been subjected to glow discharge. The samples were subsequently plunge-frozen in liquid ethane using an FEI Vitrobot Mark IV (Eindhoven, The Netherlands). These frozen grids were subsequently transferred to a 626 DH Single Tilt Cryo-Holder (Gatan, France), where they were maintained below − 180 ºC (corresponding to liquid nitrogen temperature). The grids were then transferred to TEM while being kept at liquid nitrogen temperature.

### Stability and storage of NLCs

To identify the best storage conditions for the NLCs, a stability study was conducted over a period of three months at both room temperature (RT) and 4 ºC. For this investigation, a batch of previously characterized empty nanoparticles and nanoparticles loaded with both factors was divided into six smaller sub-batches. Three of these sub-batches were stored at RT, while the other three were kept at 4 ºC. At the conclusion of each month (months 1, 2, and 3), one sub-batch from each of the storage conditions was selected and subjected to characterization. This characterization was performed using the Zetasizer Nano ZS, as detailed in Sect. “Physicochemical characterization of NLCs”.

### Cell culture

Human Umbilical Vein Endothelial Cells (HUVEC) (ATCC, Manassas, USA), were used for angiogenesis assay for testing VEGF165 bioactivity. These cells were cultivated in EGM™ Endothelial Cell Growth Medium BulletKit™ (Lonza, Basel, Switzerland) supplemented with EGM™ Endothelial Cell Growth Medium SingleQuots™ Kit (Lonza, Basel, Switzerland).

Human adipose tissuederived stem cells (ADSCs) (provided by Viscofan SA) were used for cytotoxicity and for PDGF-BB bioactivity experiments. This cells were cultured in MEM α with GlutaMAX supplement, non-nucleoside (Thermo Fisher Scientific, Waltham, USA), enriched with Fetal Bovine Serum (FBS) (Gibco, San Diego, California, USA), penicillin-streptomycin (P/S) (Gibco, San Diego, California, USA) and human Basic Fibroblast Growth Factor (hFGF-basic) (R & D Systems, Minneapolis, USA).

All cell types were incubated at 37 ºC in a 5% CO_2_ atmosphere.

### Cytotoxicity of empty NLCs

An assay was carried out to assess the toxicity of nanoparticles on human ADSCs. Cells were seeded in a 96-well plate at a concentration of 3.2 × 10^4^ cells per well and incubated overnight to allow adherence to the plate. After this period, the medium was removed, and NLC suspensions at concentrations of 0.25, 0.5, 1, 2, and 4 mg/mL were added. Additionally, Triton X-100 (SigmaAldrich, Saint Louis, USA) was added at a concentration of 1% to provide a control for cell death. The cells were exposed to the empty nanoparticles for 24 h. Then, the plate was washed with Phosphate Buffered Saline (PBS) (Gibco, San Diego, California, USA), a 1:10 solution of Cell Counting Kit-8 (CCK-8) (SigmaAldrich, Saint Louis, USA) was added and the plate was placed in the incubator. After 4 hours, the absorbance was measured at a wavelength of 450 nm with a reference of 650 nm using a plate reader (Infinite M200, Tecan, Mannedorf, Switzerland).

### Encapsulation efficiency

The EE was determined through an indirect approach, wherein the quantity of free factor, not encapsulated within the NLCs, was calculated. The explanation of the method for acquiring this sample is provided in Sect. “Preparation of nanostructured lipid carriers”. The determination of the free factor’s concentration was conducted using a commercially available EnzymeLinked Immunosorbent Assay (ELISA) specific to either VEGF165 or PDGF-BB (PeproTech, London, UK). ELISA was performed following the manufacturer’s designated protocol. Subsequently, the following equation was applied to compute the percentage of encapsulated factor:undefined

$$EE\>\left( \% \right) = {\matrix{ Initial\>amount\>of\>growth\>factor \hfill \cr - Amount\>of\>free\>growth\>factor \hfill \cr} \over {Initial\>amount\>of\>growth\>factor}}*100$$

### Sterilization

Different sterilization methods were used to determine the effectiveness in eliminating both microorganisms and spores. Consequently, two types of radiation, beta and gamma, were studied at two different doses, 12 kGy and 25 kGy.

For this purpose, the samples were sent to two different companies: Ionisos Ibérica (Cuenca, Spain) for beta radiation and Aragogamma (Barcelona, Spain) for gamma radiation. In both cases, the samples were shipped in ice-packed containers to ensure refrigeration throughout transport and irradiation, minimizing potential degradation.

For beta irradiation, upon arrival, the package was placed on a conveyor belt that transported it through the treatment area. The samples were exposed to a continuous electron beam generated by a Rhodotron TT200, moving at a speed of 3.47 m/min over a total distance of 3 m. On the other hand, for gamma irradiation, the package was placed in a container and transported via conveyor belts to the irradiation area. A Marshtype Cobalt-60 irradiator exposed the samples for the required duration to achieve the target dose. Specifically, a dose of 12 kGy was reached in 4 h and 30 min, while a dose of 25 kGy required 5 h and 15 min.

The efficacy of sterilization was investigated in accordance with Chap. 2.6.1 of the European Pharmacopoeia. First, empty nanoparticles were added to two different culture media: Fluid Thioglycollate Media (that favors the growth of anaerobic and some aerobic bacteria) and Tryptic Soya Broth (that favors the growth of fungi and some aerobic bacteria) (SigmaAldrich, Saint Louis, USA). Samples were placed at 32 ± 2 ºC and 22 ± 2 ºC, respectively, for 14 days. After this period, the medium is examined for turbidity to determine the presence or absence of bacterial growth.

Once the NLCs were received from the sterilization companies, all subsequent handling was performed under sterile conditions. This ensured that if the sterilization process had been effective, no contamination would be detected in the sterility test. Consequently, the reliability of the assay was guaranteed.

### Growth factor release profile from NLCs

The release profile of VEGF165 and PDGF-BB from NLCs was determined over 48 h. 3 mg of nanoparticles were placed in an eppendorf tube with 1 mL of 0.02 M PBS (SigmaAldrich, Saint Louis, USA) and kept in a rotor at 37 ºC. At set intervals, the tube was removed, centrifuged at 20,000 g for 15 min at 10 ºC, and the supernatant was transferred to an amicon with a 100k filter. An equivalent volume of 0.02 M PBS was added back to the tube, and the process was repeated. The supernatant transferred to the amicon may contain lipid residues from the nanoparticles. To remove these remnants, the supernatant was centrifuged again at 4000 rpm for 45 min at 10 ºC. The filtered supernatants were frozen at -20 ºC for storage.

At the end of the sampling procedure, the quantification of the factor released by the nanoparticles was carried out by ELISA, using the same procedure described in Sect. “Encapsulation efficiency”.

### Cytotoxicity of released growth factors

The cytotoxicity of the growth factors encapsulated within the NLCs was assessed according to the protocol described in Sect. “Cytotoxicity of empty NLCs”. In this specific case, the stimulus applied to the human ADSCs was derived from the supernatants of NLCs loaded with VEGF165 or PDGF-BB. Following the instructions in Sect. “Growth factor release profile from NLCs”, samples were collected from the release profiles and subsequently diluted to the desired concentrations for the assay.

### Bioactivity of the released growth factors

#### Angiogenesis assay

An angiogenesis assay (Abcam, Cambridge, UK) was conducted to evaluate the bioactivity of VEGF165 released from NLCs on HUVEC cells. Extracellular matrix solution was dispensed into each well of a 96-well plate, followed by a 1 h incubation at 37 ºC. Then, 2 × 10^4^ cells per well and VEGF165 supernatants were added. Supernatants containing the factor released from the nanoparticles were obtained as described in Sect. “Growth factor release profile from NLCs”, and dilutions were subsequently prepared to achieve the desired concentrations. Cell medium and suramin (compound that inhibits cellular vascularization, negative control) were incorporated. After 18 h of incubation, stimuli were withdrawn, followed by a washing steep. Subsequently, 100 µl of staining dye was added to each well to stain the cells and facilitate de observation of their arrangement. After an additional 1 h incubation, vessel formation was observed under fluorescence microscopy.

The images were subjected to a comprehensive analysis using specialized software provided by Wimasis (Córdoba, Spain). This analysis yielded a range of distinct parameters related to cellular vascularization, including covered area, total tubes, total tube length, total branching points and total loops.

#### Wound healing assay

PDGF-BB bioactivity was assessed by wound healing assays on human ADSCs. Wound healing inserts (Abcam, Cambridge, UK) were carefully placed in each well of a 24-well plate thus, separating the well surface into two halves. A cellular density of 5 × 10^4^ cells per well was introduced on either side and incubated for 24 h at 37 ºC with 5% CO_2_. After incubation, inserts were removed and wells were thoroughly washed with PBS. Subsequently, different concentrations of PDGF-BB were added from the nanoparticle supernatants. These supernatants were obtained as described in Sect. “Growth factor release profile from NLCs” and then diluted to obtain the desired concentrations. The plate was then positioned in the Cytation1 (BioTek, Winooski, USA), capturing images at 20 min intervals over 48 h. Gen5 3.10 software was employed to analyze all images acquired by Cytation1 with the objective of quantifying wound closure values.

### Statistical analysis

The data obtained from the study were subjected to analysis employing SPSS Statistics 28 software, with the presentation of results in the format of mean values accompanied by their corresponding standard deviations (SD). The normal distribution of the sample sets was assessed using the Kolmogorov-Smirnov test. Furthermore, the homogeneity of variance was evaluated through the application of the Levene test. Ultimately, the means were compared employing a one-way analysis of variance (ANOVA), followed by the implementation of post-hoc tests, namely the Bonferroni or Tamhane procedures. Significant differences were defined as those with *p* < 0.05.

## Results and discussions

### Characterization of NLCs

**Fig. 1:**
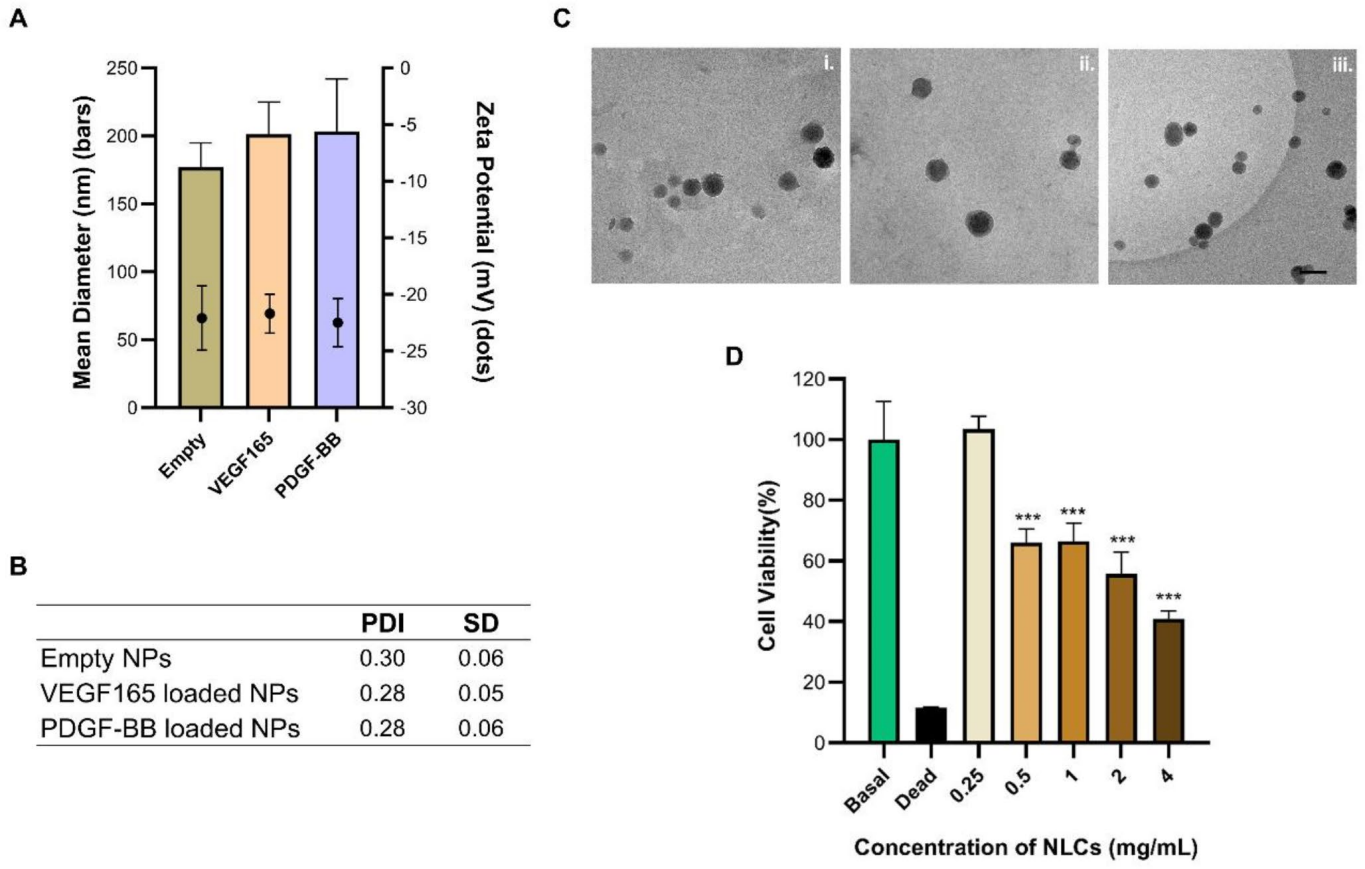
Physicochemical characterization of NLCs. (**A**) Size (bars) and zeta potential (dots) of empty and loaded with VEGF165 and PDGF-BB NLCs. Each value represents the mean ± standard deviation (SD). *n* ≥ 3. (**B**) Table with mean PDI values and SD of empty and loaded VEGF165 and PDGF-BB NLCs. *n* ≥ 3. (**C**) Cryo-TEM images of NLCs showing: **(i)** empty, **(ii)** VEGF165 loaded NLCs and **(iii)** PDGF-BB loaded NLCs. Scale bar: 200 nm. (**D**) Cell viability of empty NLCs on human adipose derived mesenchymal cells, expressed as a percentage according to the CCK-8 assay. *** *p* < 0.001. vs. basal condition. *n* = 3

The measurement of nanoparticle properties is of great importance, as variations in these characteristics can cause different physical and chemical behavior [14]. Figure 1A illustrates the results concerning the particle size and zeta potential of NLCs, including both empty ones and those loaded with VEGF165 or PDGF-BB. Notably, all nanoparticles exhibited diameters ranging from 175 nm to 210 nm. The literature has documented a broad spectrum of NLCs sizes, with reports indicating dimensions below 100 nm [31] as well as exceeding 300 nm [32]. However, a comprehensive review by A. Gordillo-Galeano et al. established the typical particle size range for NLCs to be between 100 nm and 400 nm. Furthermore, this review highlighted that the most prevalent values consistently ranged within the range of 150 nm to 250 nm [33]. This indicates that the size of the NLCs is consistent with the expected standard dimensions, suggesting a good uniformity and quality in their production.

Additionally, it is noteworthy that NLCs typically exhibit a negative zeta potential, with values that can extend to approximately − 40 mV [34]. High positive or negative values enhance the repulsive forces between nanoparticles, thereby increasing their suspension stability. In several studies involving encapsulation with NLCs, comparable values have been observed [32, 35]. In this study, the zeta potential of the NLCs was around − 22 mV, indicating highly negative values that suggest stable suspension behavior.

Furthermore, the PDI, a measure of sample uniformity, was subsequently assessed. A PDI value ≤ 0.30 is widely acknowledged to be indicative of sample homogeneity. The PDI values obtained from the formulation of both empty NLCs and those loaded with the factors are presented in Fig. 1B. In each case, the PDI values ranged from 0.28 to 0.30, consistent with results reported in previous scientific studies [36, 37].

Validation of nanoparticle morphology was then carried out using cryo-TEM. This technique is commonly used for the morphological elucidation of NLCs [38, 39]. This is due to their increased sensitivity and susceptibility to degradation during sample preparation. Figure 1C shows the cryo-TEM images showing spherical particles in all three categories of NLCs, including empty NLCs and those encapsulating VEGF165 or PDG-FBB.

In addition, it is important to determine whether the produced nanoparticles exhibit toxicity to cells; for this purpose, a cytotoxicity study was performed and the results are presented in Fig. 1D. Different concentrations of NLCs, ranging from 0.25 mg/mL to 4 mg/mL, were tested. As a result, only the lowest concentration, 0.25 mg/mL, did not cause a decrease in cell viability. Above this value, the other concentrations tested showed toxic effects on the cells. At concentrations of 0.5 and 1 mg/mL, cell viability decreased to 70%, at 2 mg/mL to 60% and at 4 mg/mL to 40%. The surfactants in the NLC composition can cause irritation and have some toxicity [40], which may explain these results. As the concentration of nanoparticles increased, the amount of surfactant also increased, leading to a more pronounced toxic effect.

Finally, it is worth noting that the NLCs were prepared using the emulsification-ultrasonication technique. This method is particularly relevant as it does not require specialized equipment or the use of excessively high energy inputs [41]. All of these factors facilitate the future scaling-up process at an industrial level.

### Three-month stability of NLCs

**Fig. 2:**
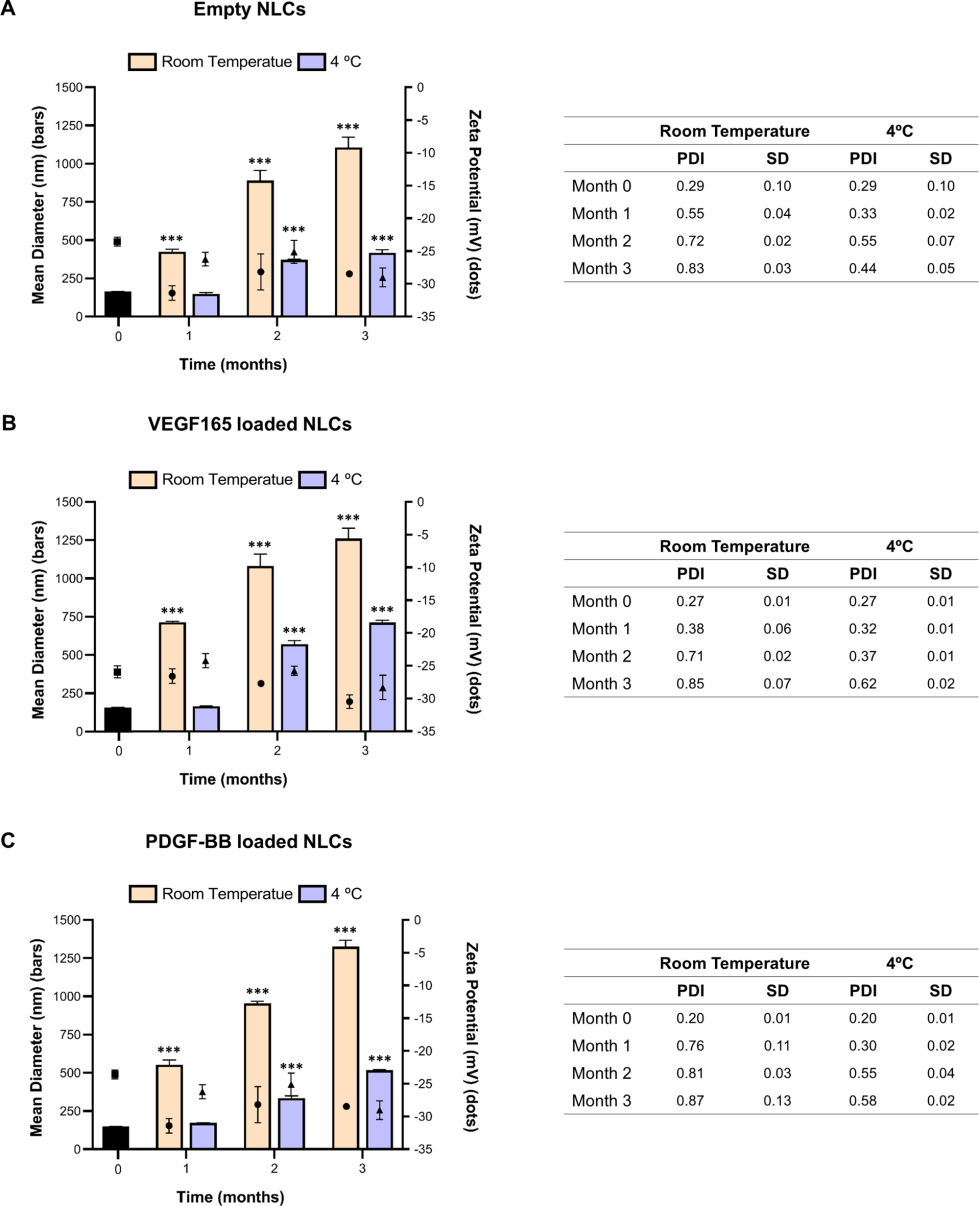
Stability of (**A**) empty NLCs, (**B**) NLCs loaded with VEGF165, and (**C**) NLCs loaded with PDGF-BB over three months under two different storage conditions: room temperature and 4 ºC. Particle size (bars) and zeta potential (dots) are shown, with black bars representing the initial condition, orange bars corresponding to room temperature storage, and blue bars indicating storage at 4 ºC. Each value represents the mean ± standard deviation (SD) (*n* ≥ 3). A table with the mean PDI values and SD of nanoparticles is included. *** *p* < 0.001 vs. month 0. *n* = 3

The stability of NLCs is crucial, as they have a tendency to increase in particle size and form aggregates [42]. This leads to a reduction in the specific surface area of the nanoparticles, resulting in a slower release rate, decreased drug loading capacity and reduced nanoparticle-cell interaction [43]. Such changes could be detrimental, as the concentration of the factors may fluctuate outside the therapeutic window, thereby compromising the intended efficacy. Consequently, a stability study was conducted on both the empty NLCs and the NLCs loaded with VEGF165 and PDGF-BB, under two conditions: RT and 4 ºC. The findings derived from this investigation are presented in Fig. 2. It is clear that all NLCs types, whether loaded with growth factors or not, show identical behavior during storage. For nanoparticles stored at RT, significant differences were observed from the first month, with both size and PDI increasing to values above 1000 nm and 0.83, respectively. Conversely, nanoparticles stored at 4 ºC remained stable in size and PDI during the first month. However, during the second and third months, an increase in size and PDI is observed, reaching values of 400 nm and 0.55 respectively. This behavior is consistent with the research conducted by Z. Jafarifar, who investigated the stability of NLCs at 4 ºC, 25ºC and 37 ºC, finding that higher temperatures resulted in an increase in nanoparticle size [44]. Therefore, the best approach is to store the nanoparticles at 4 ºC for one month to maintain all their properties stable. This practice avoids the need to prepare a new batch each time it is required, facilitating large-scale production and ensuring a continuous supply.

### Loaded NLCs encapsulation efficiency

The EE is a quantitative measure denoting the fraction of the factor enclosed within nanoparticles relative to the total amount of the factor employed for their manufacturing. The EE of VEGF165 and PDGF-BB-loaded NLCs was high, with values of 87 ± 4.23% and 95 ± 2.84%, respectively. Scientific literature has reported that the EE typically exceeds 70% when bioactive compounds are encapsulated within NLCs [33]. The high EE values can be attributed to the internal structure of the NLCs. These are characterized by a less rigid matrix compared to other lipid nanoparticles, such as solid lipid nanoparticles (SLNs) [45], allowing them to encapsulate a greater amount of growth factor. This ensures that a greater amount of VEGF165 or PDGF-BB is available at the target area, as the loss of drug during the encapsulation process is minimized.

### Selection of the sterilisation method

The achievement of sterility is crucial for the clinical applicability of the synthesized nanoparticles. Consequently, various sterilization methods were considered and analyzed to assess their feasibility. Filtration was excluded since some bacteria range in size from 100 nm to 200 nm, which overlaps with the size of the nanoparticles [46]. On the other hand, dry heat sterilization requires temperatures of 150 ºC or 160 ºC [47], making it unfeasible, as the lipids would melt and the stability of the factors would be compromised. Similarly, moist heat sterilization or autoclaving requires temperatures of 121 ºC [48], leading to the same issues for the NLCs. For these reasons, ionizing radiation was chosen, as it is known to cause minimal damage to the sample without a significant increase in temperature [23]. Additionally, in both cases, it is possible to ship pallets containing a large quantity of nanoparticles, making industrial-scale production feasible.

**Fig. 3:**
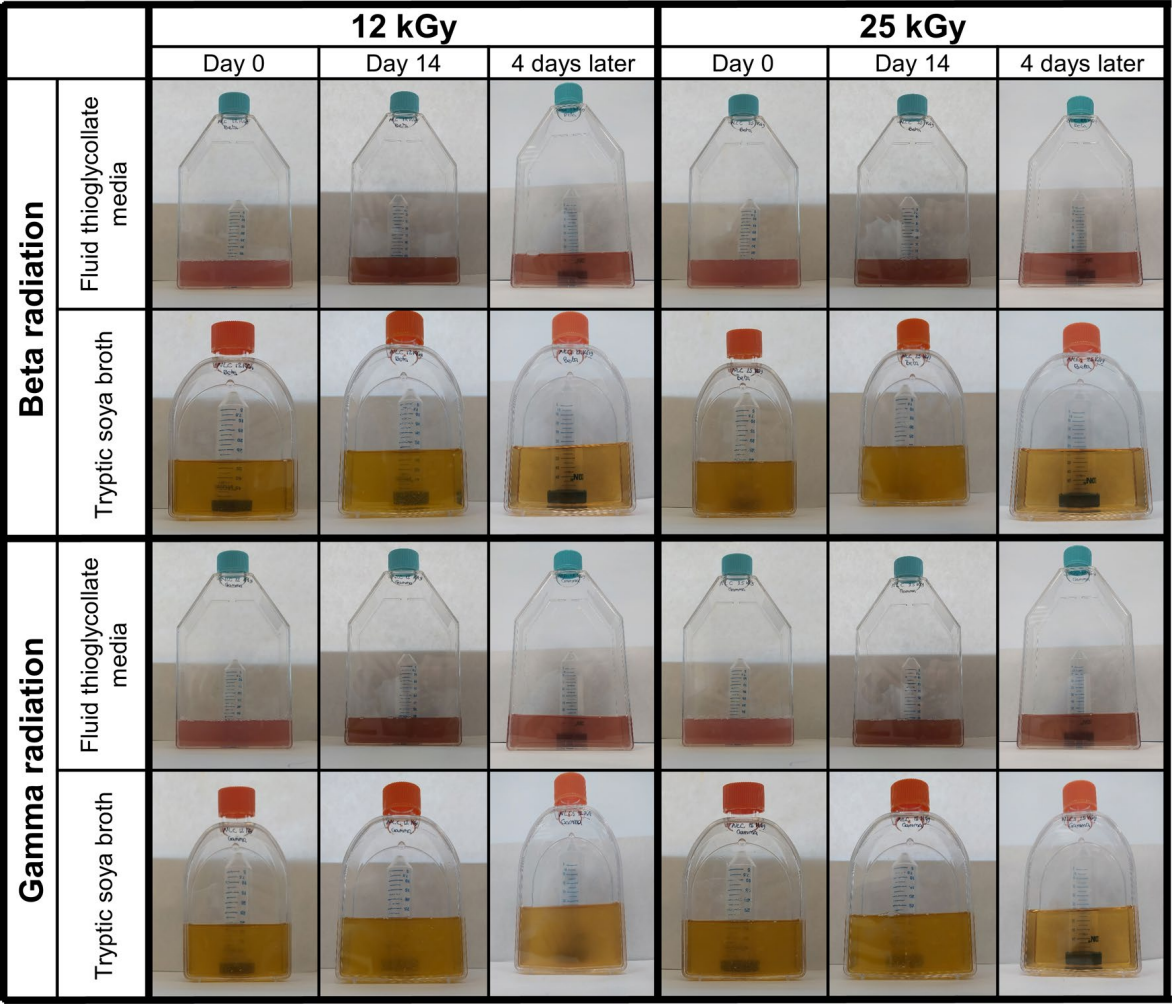
Sterility evaluation of empty NLCs irradiated with beta and gamma radiation at doses of 12 kGy and 25 kGy according to the Chap. 2.6.1 of the European Pharmacopoeia

Therefore, sterilization procedures were performed using both beta and gamma radiation at doses of 12 kGy and 25 kGy. According to the EMA’s sterilization guidelines, a reference dose of 25 kGy is established; however, other doses may be used as long as they prove effective in eliminating all microorganisms [49]. The effect of both types of radiation is dose dependent, meaning that higher doses have a greater effect on the irradiated product [50, 51]. Consequently, the study also included a lower dose of 12 kGy to determine if it could effectively sterilize the batch of nanoparticles while producing fewer adverse effects. Afterwards, the successful sterilization of nanoparticles by irradiation was tested in accordance with Chap. 2.6.1 of the European Pharmacopoeia (Fig. 3). The introduction of nanoparticles into the culture medium induced turbidity from the very beginning. Consequently, in accordance with the European Pharmacopoeia protocol, an aliquot of the turbid culture medium was taken and transferred into fresh culture medium, where it was incubated for 4 days. At the end of this period, in general, no bacterial proliferation was observed. However, turbidity was observed in the tryptic soya broth medium containing nanoparticles irradiated with gamma radiation at a dose of 12 kGy, indicating the ineffectiveness of the sterilization process.

Sterilization by beta and gamma radiation at a dose of 25 kGy proved to be effective in the eradication of all bacterial strains, including both anaerobic and aerobic varieties. This result is in accordance with expectations, as the standard dose commonly used for nanoparticle sterilization is set at 25 kGy [52]. However, when the 12 kGy dose was used, different behaviors were observed. In the case of beta radiation, no bacterial growth was observed in either culture medium. Conversely, when gamma radiation was used, turbidity appeared in the tryptic soya broth medium, indicating bacterial growth. Both types of radiation have a very similar mechanism, although beta radiation uses a higher amount of energy [53]. In addition, gamma radiation has a dose-dependent effect, where higher doses result in a more pronounced irradiation effect [54]. Both the difference in energy levels and the dose-dependent effect may explain the lack of sterilization in the nanoparticle batch at the 12 kGy dose. As a consequence, in subsequent experiments, the 12 kGy gamma radiation condition was discarded.

### Characterization of NLCs after irradiation

**Fig. 4:**
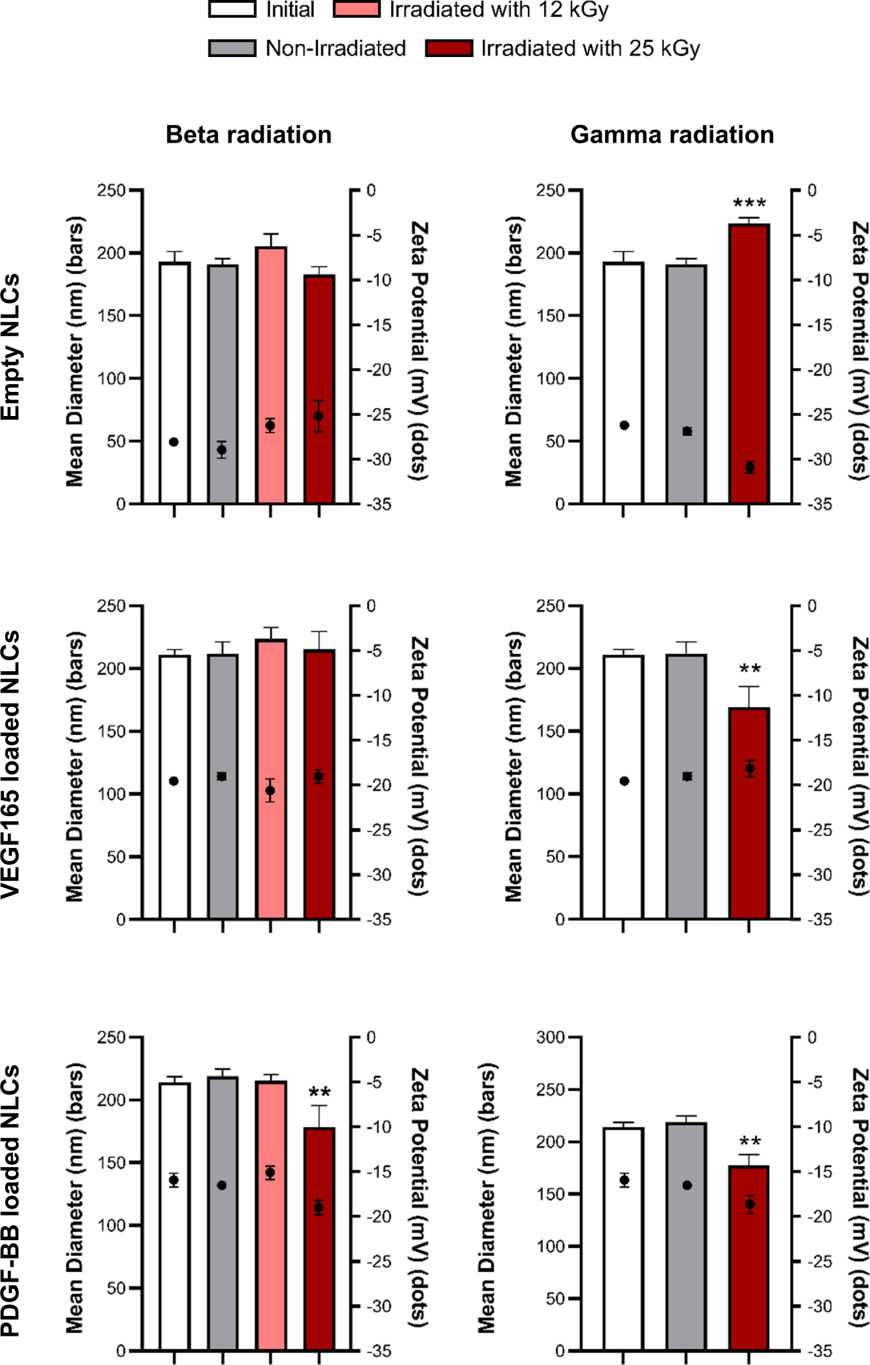
Physicochemical characterization of empty and VEGF165 and PDGF-BB-loaded NLCs before and after irradiation with beta and gamma radiation. Size (bars) and zeta potential (dots) values. Each value represents the mean ± standard deviation (SD). ** *p* < 0.01 and *** *p* < 0.001. vs. initial values. *n* = 3

The use of ionizing radiation, including beta and gamma radiation, can have a detrimental impact on the material to which it is applied. This damage results primarily from the introduction of free radicals and ions, which have the ability to compromise the integrity of DNA, lipids, proteins and other essential components [54]. Consequently, it is imperative to evaluate the integrity of NLCs after the sterilisation processes. In the first stage, an assessment was carried out to investigate any changes in the physicochemical and structural characteristics of the nanoparticles. For this purpose, nanoparticles immediately after preparation, nanoparticles sent to the irradiation facility but not irradiated (taking into account possible stability issues during transport) and nanoparticles irradiated at specific doses were analysed. The resulting data are presented in Fig. 4; Table 1.

**Table 1:** Physicochemical characterization of empty and VEGF165 and PDGF-BB-loaded NLCs before and after irradiation with beta and gamma radiation. Mean polydispersity index (PDI) values and standard deviation (SD)

	Beta radiation		Gamma radiation	
	Mean PDI	SD	Mean PDI	SD
	Empty NLCs			
Initial	0.27	0.03	0.21	0.01
Non-irradiated	0.24	0.10	0.21	0.01
12 kGy	0.22	0.05		
25 kGy	0.23	0.16	0.22	0.02
	NLCs loaded with VEGF165			
Initial	0.23	0.01	0.29	0.07
Non-irradiated	0.20	0.03	0.27	0.06
12 kGy	0.19	0.06		
25 kGy	0.22	0.04	0.34	0.04
	NLCs loaded with PDGF-BB			
Initial	0.22	0.05	0.24	0.02
Non-irradiated	0.25	0.04	0.27	0.02
12 kGy	0.26	0.05		
25 kGy	0.27	0.08	0.34	0.12

The results of the study showed different effects on NLCs depending on both the type and dose of radiation to which they were exposed.

Regarding the beta radiation, exposure to 12 kGy did not modify any of the nanoparticle properties, as evidenced by the lack of significant differences among groups. However, significant differences were observed only in PDGF-BB-loaded NLCs when sterilized with 25 kGy of beta radiation; the remaining NPs were unchanged. Conversely, for gamma radiation, the 12 kGy dose was considered unsuitable as it failed to achieve complete sterilization of the nanoparticle batch. On the other hand, when exposed to 25 kGy of gamma radiation, significant differences were observed in both empty nanoparticles and nanoparticles loaded with VEGF165 or PDGF-BB.

A study by Y. S. Tapia-Guerrero et al. concluded that the use of gamma radiation can induce changes in nanoparticles in a dose-dependent manner [56]. These findings are supported by various studies which have observed that high doses of gamma radiation can modify nanoparticle size, PDI and zeta potential [56, 57]. Additionally, O. Maksimeko et al. irradiated nanoparticles with both gamma and beta radiation and concluded that gamma radiation induced changes in their physicochemical properties, whereas beta radiation had no effect [59]. The difference may be attributed to the greater effect of gamma radiation on the sample compared to beta radiation [60]. This study supports the conclusion that beta radiation induces minimal changes in properties while gamma radiation increases these alterations.

### Release profile before and after sterilization

**Fig. 5:**
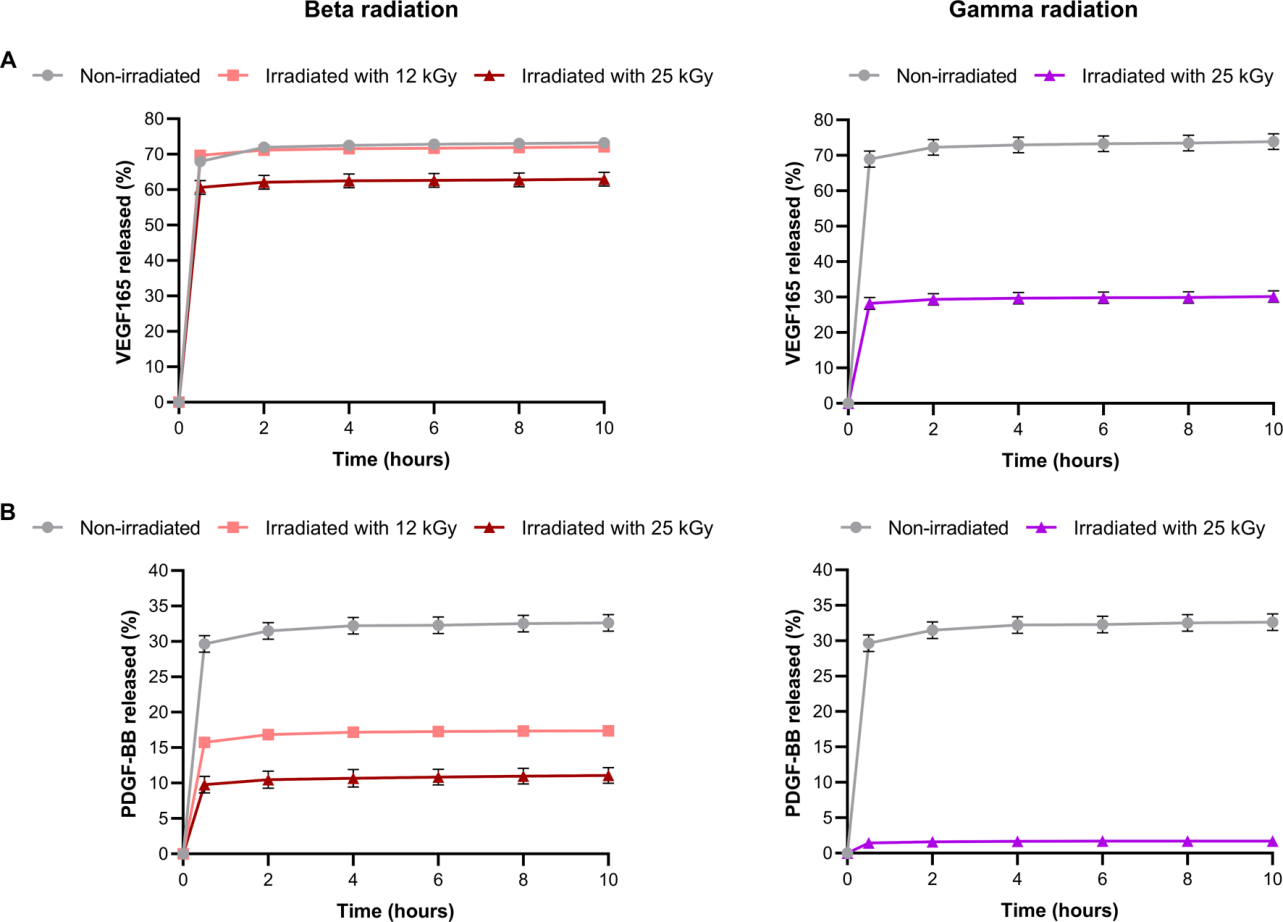
Release profiles of NLCs loaded with (**A**) VEGF165 and (**B**) PDGF-BB, without irradiation and irradiated with beta and gamma radiation, analyzed by ELISA expressed as a percentage of the total VEGF165 or PDGF-BB released. *n* = 3

The way in which the growth factors are released from the nanoparticles will influence their properties, including bioavailability, clearance and stability, among other parameters [60]. The type of release will affect clinical treatments, potentially leading to different approaches in administration, dosage and related aspects. Additionally, irradiation may alter both the form and amount of factor released, leading to a completely different release profile from that of non-irradiated nanoparticles. Therefore, it is necessary to perform a study to determine the release profiles of unsterilized and sterilized nanoparticles, taking into account the potential impact of the irradiation process on the release rate of these factors. The results of the release profiles are presented in Fig. 5.

Both VEGF165 and PDGF-BB show a rapid release from the NLCs over a short period of time. This behavior is characteristic of NLCs and this effect may be enhanced by the reduced dimensions of the nanoparticles. It is well known that the rate of release of encapsulated compounds from a nanoparticle is inversely proportional to its diameter, with smaller nanoparticles eliciting faster release kinetics [61]. A similar burst-type release pattern was observed for NLCs in a study by V.L. Beraldo-Araújo et al. [34]. Therefore, it is essential to analyze the amount of growth factor released from nonirradiated nanoparticles compared to the initially encapsulated amount. In this context, there are differences between NLCs loaded with VEGF165 and those loaded with PDGF-BB. In the former, approximately 75% of the originally encapsulated factor was released, whereas in the latter, 30% of the total encapsulated factor was found to be released. Different independent studies confirm that the majority of NLCs achieve a release ≥ 65% of the total encapsulated protein [63–65]. The limited release of PDGF-BB-loaded NLCs, which was 30%, can be attributed to the potential denaturation of the growth factor. As shown in Figure S1A of the Supplementary Material, the nano differential scanning calorimetry (nano-DSC) analysis of PDGF-BB shows a prominent peak at 71 ºC. However, it is noteworthy that the onset of this peak takes place at a lower temperature, specifically at 60 ºC, and concludes at 85 ºC. During the nanoparticle synthesis process, it is necessary to heat the lipids to approximately 65 ºC for complete melting. Consequently, there is a possibility that some PDGF-BB undergoes denaturation prior to the formation of NLCs, making its detection impossible. Consequently, as shown in Figure S1B of the supplementary materials, a sample of PDGF-BB that had been previously heated was analyzed. It was confirmed that exposure to high temperatures affects PDGF-BB, leading to its denaturation and preventing its complete detection by ELISA.

Looking at the behavior of the factors released by the sterilized nanoparticles, in the case of VEGF165, when exposed to beta radiation at 12 kGy, there was no difference compared to the nanoparticles that had not been irradiated. However, when the 25 kGy dose was applied, the nanoparticles released 10% less factor than the non-irradiated nanoparticles. A similar trend was observed for PDGF-BB. When exposed to 12 kGy of beta radiation, the release of the factor is reduced by 50% compared to the non-irradiated nanoparticles, and this reduction increases to one third when exposed to 25 kGy of beta radiation. This effect can be attributed to the dose-dependent nature of beta radiation, where an increase in the dose applied leads to an intensification of the effect, causing greater damage to the growth factor-loaded nanoparticles [66]. With regard to gamma radiation, irradiation of VEGF165-loaded NLCs at 25 kGy results in a decrease of almost 50% in the released factor. Conversely, for PDGF-BB, a greater decrease was observed, with only 3% of the factor detected. In a study conducted by A. Omar et al., in which ciprofloxacin nanoparticles were irradiated with gamma radiation, it was observed that the amount of drug released was reduced [67]. Furthermore, another experiment confirmed that this reduction was dose-dependent, with higher doses causing greater damage [68]. However, a trial conducted by I. Dománska et al. emphasized that the effect of beta radiation on nanoparticles was minimal, only slightly decreasing the release rate [69].

This difference between the effects of the two types of radiation can be attributed to the greater impact of gamma radiation on the sample compared to beta radiation [59]. Consequently, it was observed that while beta radiation induced minimal changes in the physicochemical properties of the NLCs, it could penetrate and affect the growth factors. In contrast, gamma radiation, which is a more aggressive method, induces changes in both the physicochemical properties of the nanoparticles and the encapsulated factors. These changes may lead to modifications in the molecular structure of the growth factors, thereby affecting their bioactivity.

### Cytotoxicity of VEGF165 and PDGF-BB released from NLCs

**Fig. 6:**
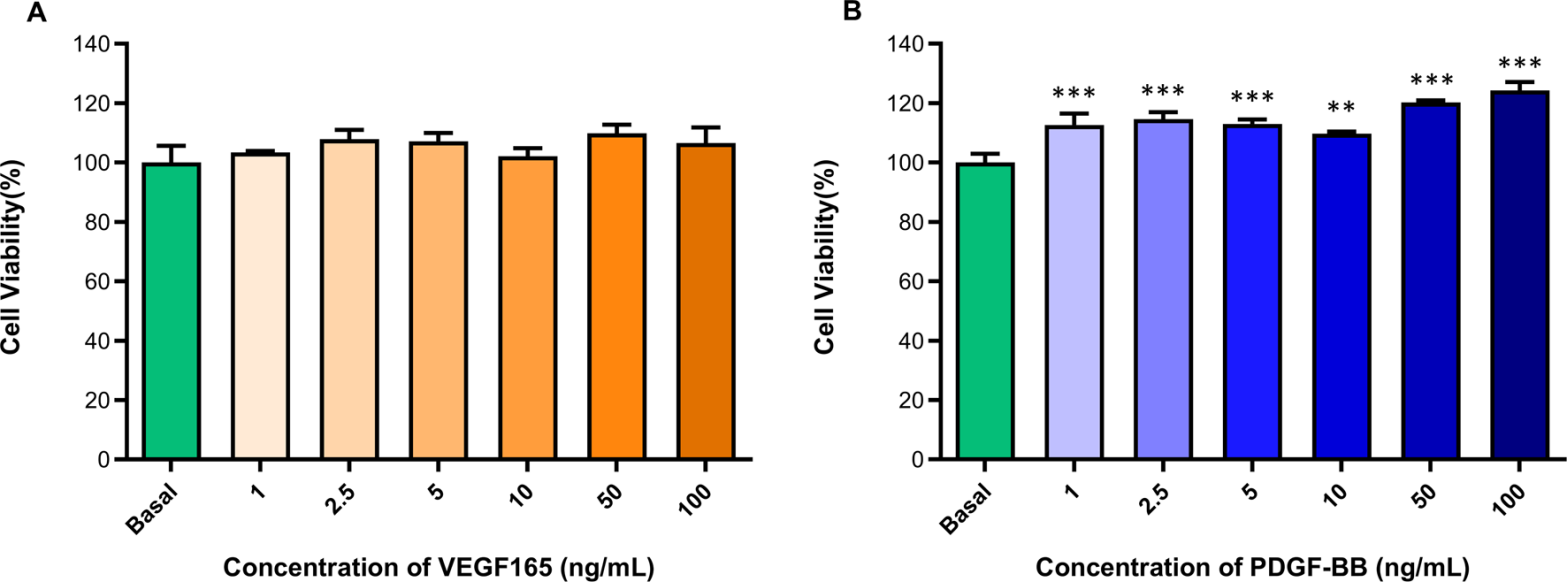
Cell viability expressed in percentage by means of CCK-8 of (**A**) VEGF165 released from NLCs and (**B**) PDGF-BB released from NLCs on human ADSCs. ** *p* < 0.01 and *** *p* < 0.001. vs. basal condition. *n* = 3

Toxicity evaluation of the growth factors once they are released from the nanoparticles is crucial as excessively high concentrations can be harmful to cells. For this purpose, a CCK-8 assay was performed on human ADSCs using supernatants from NLCs with the results shown in Fig. 6. The concentrations tested ranged from 1 ng/mL to 100 ng/mL for both growth factors, and neither VEGF165 nor PDGF-BB exhibited toxic effects. However, significant differences were observed for PDGF-BB, as all concentrations increased cell viability compared to cells treated with culture medium. Since this growth factor promotes cell proliferation [70], this results in higher cell numbers, which leads to increased cell viability. This finding is supported by several studies on both VEGF16^5^ [70, 72] and PDGF-BB [73, 74]. Taken together, these results confirm that the concentrations of growth factors released from the nanoparticles do not reach the minimum toxic dose. Consequently, both VEGF165 and PDGF-BB can be used at these concentrations without inducing toxic effects. However, it is essential to investigate the therapeutic window to determine the most effective dose for use in future treatments.

### Bioactivity of encapsulated VEGF165 and PDGF-BB, before and after sterilization

#### Angiogenesis assay

**Fig. 7:**
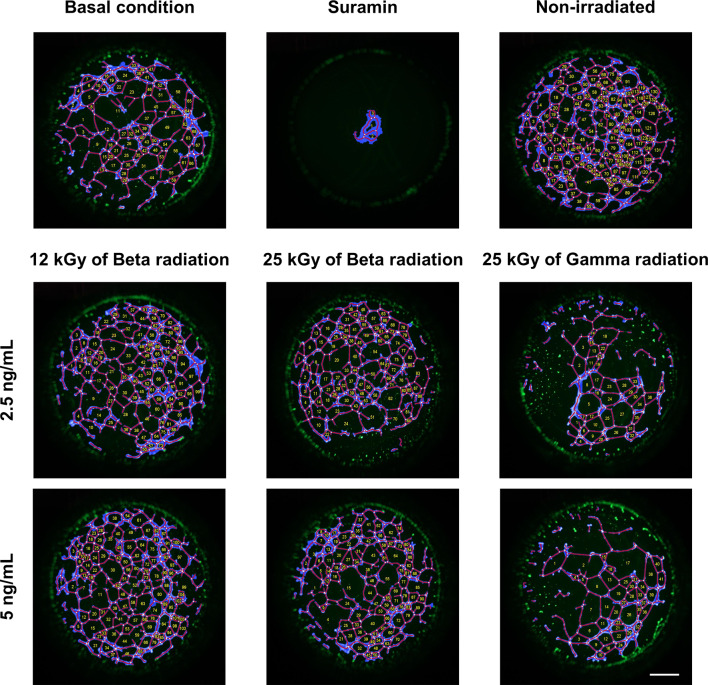
Analyzed images of the angiogenesis assay of VEGF165 derived from NLCs. Basal condition, suramin (negative control), non-irradiated NLCs, NLCs irradiated with 12 kGy of beta radiation, NLCs irradiated with 25 kGy of beta radiation and NLCs irradiated with 25 kGy of gamma radiation. Employing two different concentration: 2.5 ng/mL and 5 ng/mL. Showing: in blue the covered area, the tubes in red, the branches as white dots and the vessels formed with a yellow number. Scale bar: 1000 μm

Due to the instability nature of growth factors, they can be denatured by irradiation. Therefore, it is important to check for any changes in the bioactivity of the active molecules. Since VEGF165 promotes the formation of new vascular structures, an angiogenesis assay was performed to evaluate the biological efficacy of the encapsulated factor within the NLCs. These experiments were conducted on HUVEC cells, as this is a well-established model for studying in vitro angiogenesis [75], and different studies have demonstrated its effectiveness in this field [75, 76]. In a preliminary assessment, the release of different concentrations of VEGF165 from NLCs, ranging from 1 ng/mL to 10 ng/mL, was studied to identify the concentrations with maximal efficacy. These results are shown in Figure S2 and S3 in the Supplementary Appendix. The graph shows that administration of 1 ng/mL resulted in significant differences in the total number of branching points, total tubes and total loops. However, no differences were observed in the analysis of covered area or tube length. Conversely, both the 2.5 ng/mL and 5 ng/mL doses enhanced angiogenesis in all parameters. Nevertheless, a more pronounced effect was observed with the 2.5 ng/mL dose. Finally, the 10 ng/mL dose did not result in any improvement in cellular vascularization. Therefore, to evaluate the effect of VEGF165 released from NLCs after irradiation, concentrations of 2.5 and 5 ng/mL were used. The resulting images were subjected to a rigorous analysis, as shown in the Fig. 7.

**Fig. 8:**
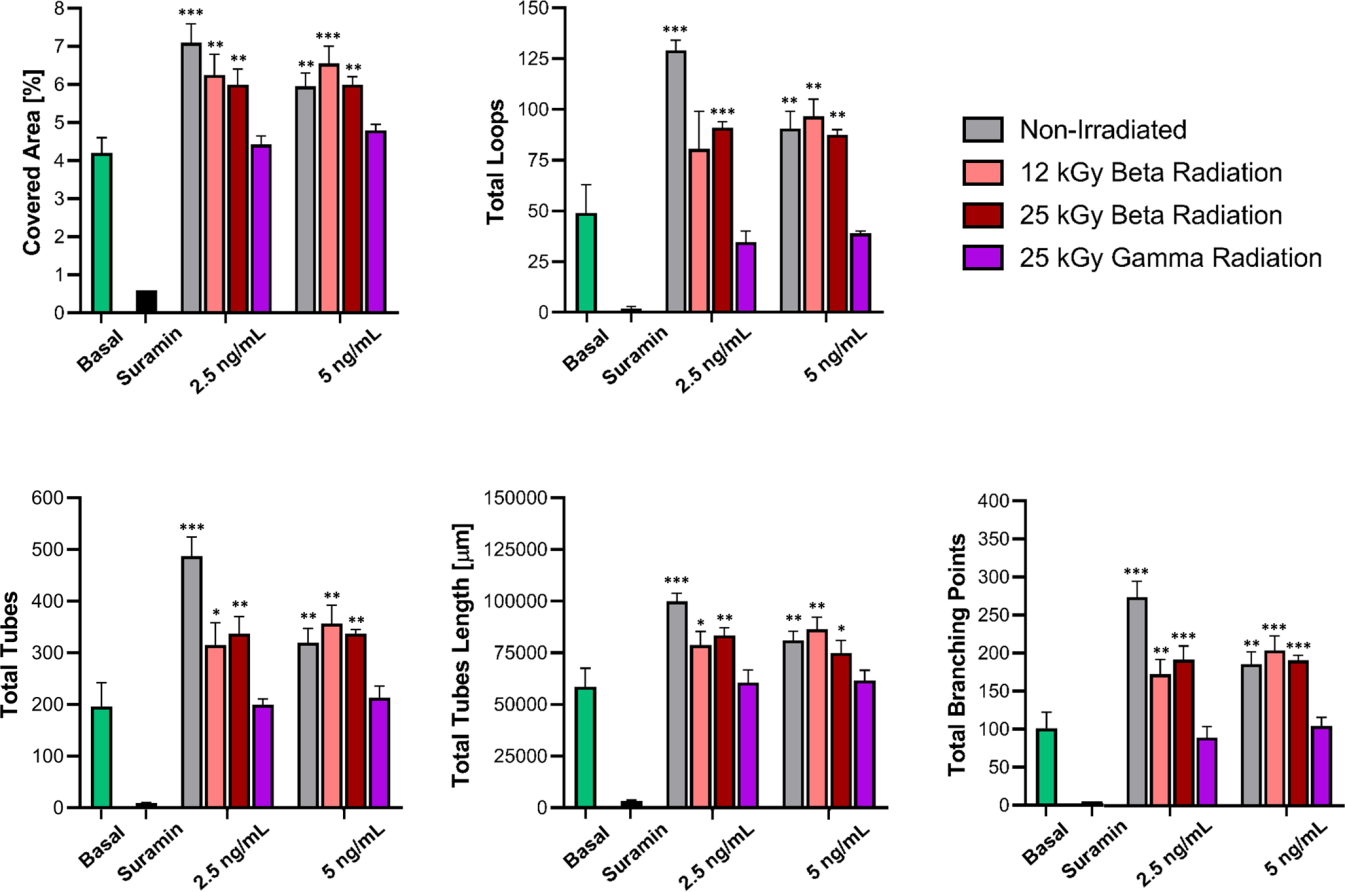
Quantitative parameters analysis on the angiogenesis assay. Comparison of the effect on HUVEC cells of medium of culture (green bars), suramin as negative control (black bars), VEGF165 released from non-irradiated NLCs (gray bars), NLCs irradiated with 12 kGy (pink bars) and 25 kGy (red bars) of beta radiation and NLCs irradiated with 25 kGy of gamma radiation (purple bars). * *p* < 0.05, ** *p* < 0.01 and *** *p* < 0.001 vs. basal. *n* = 3

Preliminary analysis revealed differences between the conditions tested. In particular, it was observed that the addition of culture medium, in the absence of any external stimulus, induced an angiogenic effect. In contrast, the use of suramin effectively removed any potential angiogenic response. However, observable differences arise upon the addition of released VEGF165. Therefore, in order to obtain conclusive results, it is essential to quantify these data. Figure 8 shows the quantitative assessment of the acquired images.

Several studies have focused on the study of angiogenesis, although the parameters examined may vary. Some studies concentrate only on the analysis of the covered area [77], while others focus on the total length of the tubes [78]. In some cases, the number of branching points is analyzed in addition to these two parameters [79]. The more parameters analyzed, the more reliable the results. In this particular study, it was decided to examine five different parameters.

The five parameters consistently show similar trends. In particular, by using the concentration of 2.5 ng/mL, it is observed that VEGF165 from non-irradiated NLCs has a higher activity than that from irradiated NPs. However, the factor released from NLCs irradiated with 12 kGy and 25 kGy of beta radiation still shows significant differences compared to the baseline condition, thereby retaining its biological activity. Conversely, VEGF165 released from NLCs irradiated with 25 kGy of gamma radiation completely loses its angiogenic effect and shows no differences from baseline.

On the other hand, when a concentration of 5 ng/mL is used, a different behavior is observed. VEGF165 derived from non-irradiated NLCs shows a decrease in bioactivity, although it remains biologically active. This behavior is attributed to the optimal concentration of VEGF165 being 2.5 ng/mL, as shown in Figure S3 in the supplementary material. Consequently, when this concentration is modified and 5 ng/mL is used, there is a slight decrease in bioactivity. In contrast, when the results for VEGF165 from NLCs sterilized with 12 kGy and 25 kGy of beta radiation are examined, biological activity is maintained and angiogenesis is observed. Finally, the analysis of VEGF165 released from NLCs irradiated with 25 kGy of gamma radiation shows no significant differences with respect to the baseline, indicating a loss of angiogenic effect of the factor.

As a result, the use of gamma radiation leads to a complete loss of the biological activity of VEGF165 encapsulated within the NLCs. In contrast, when beta radiation is used to sterilize the nanoparticles, there is a slight reduction in the angiogenic effect, but the factor maintains its biological activity. This discrepancy can be attributed to the different effects of beta and gamma radiation, the former having a greater impact on the NLCs and their encapsulated factors [59]. Nevertheless, since beta radiation consists of a highenergy electron beam, it also affects the VEGF165 encapsulated in the NLCs, which explains the reduction in bioactivity observed [22].

#### Wound healing assay

**Fig. 9:**
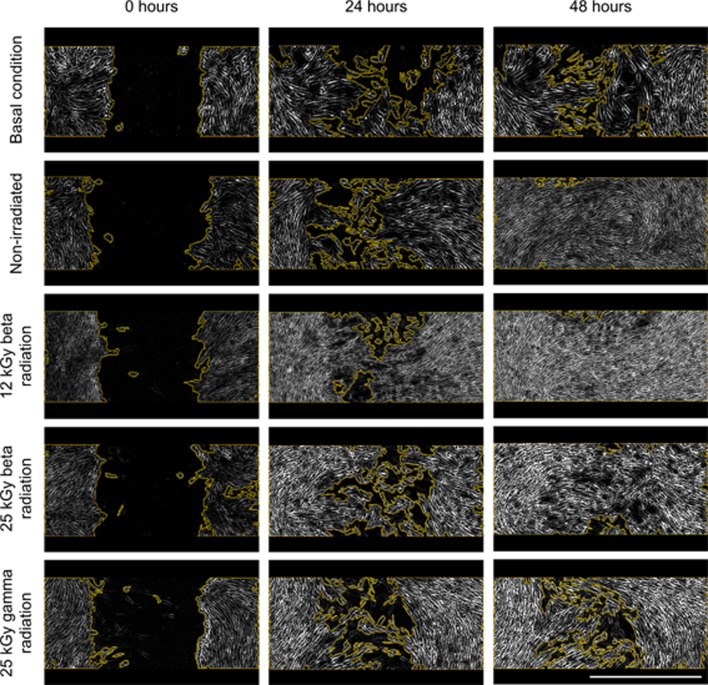
Wound healing assay on human ADSCs applying different stimulus at 0, 24 and 48 h of exposure. Using basal condition (culture medium) and PDGF-BB from NLCs that were non-sterilized, sterilized with 12 kGy and 25 kGy of beta radiation and 25 kGy of gamma radiation. The yellow line delimits the area of cells detected in the image. Scale bar 1000 μm

PDGF-BB, known for its capacity to stimulate cell migration and proliferation, was evaluated through a wound healing assay. This experiment was conducted on human ADSCs, as they have been shown to be useful in bone and cartilage regeneration models due to their ability to differentiate into both osteoblasts and chondrocytes [80–83]. First, an experiment was carried out with PDGF-BB concentrations ranging from 10 ng/ml to 100 ng/ml. The aim was to determine the concentration at which the factor showed maximum biological activity. As shown in Figure S4 of the supplementary material, this peak is reached at a concentration of 50 ng/mL. Consequently, this concentration was used in the subsequent assay, both for non-irradiated nanoparticles and for those exposed to beta and gamma radiation (Fig. 9). The images illustrate the differences in the wound closure process depending on the stimulus applied.

**Fig. 10:**
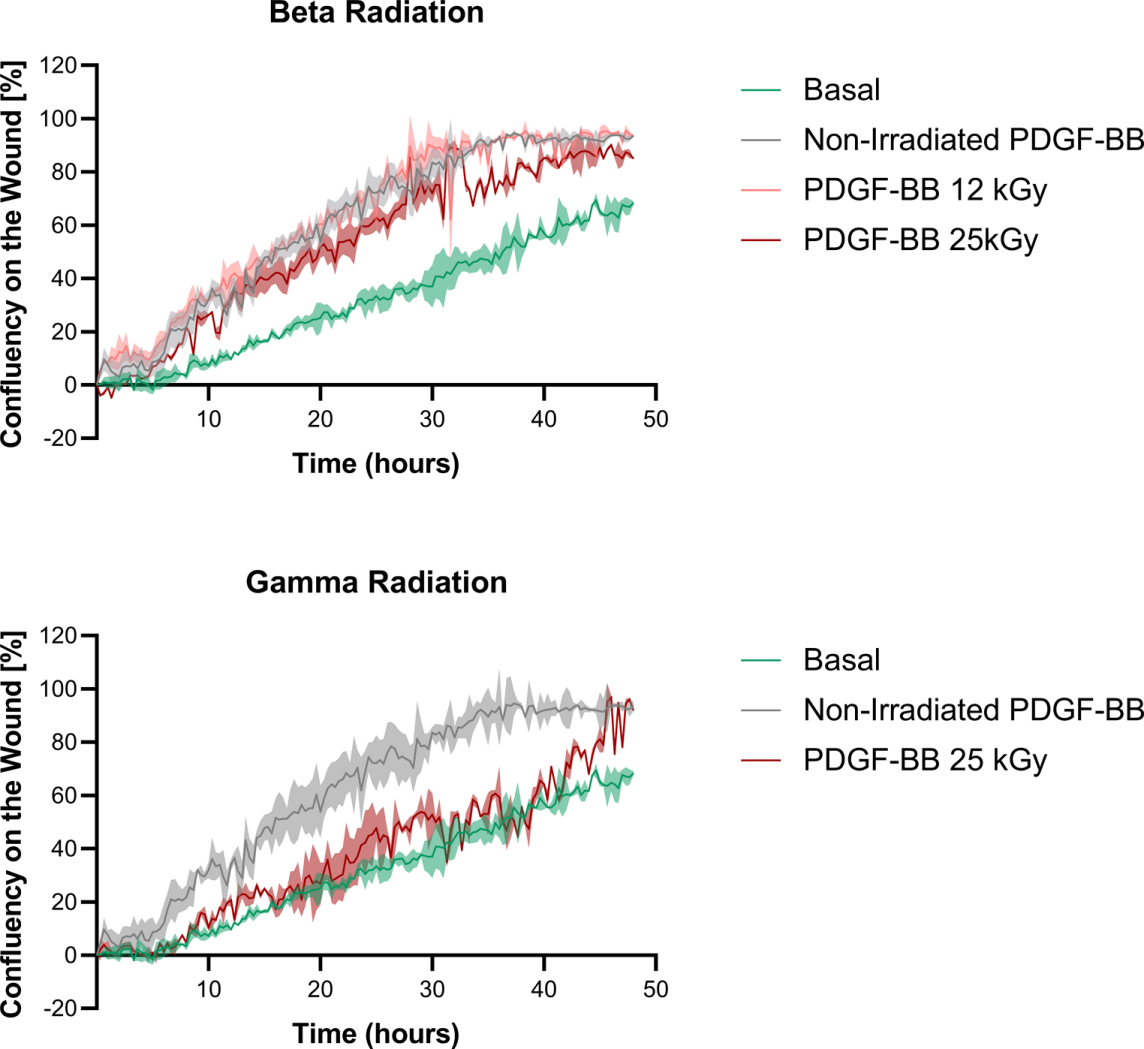
Quantitative analysis of wound healing assay on human ADSCs employing the supernatants of PDGF-BB-loaded NLCs non-irradiated and irradiated with beta radiation and gamma radiation. The standard deviation (SD) of the values is represented by the shaded area. *n* = 3

The addition of the culture medium, as well as the PDGF-BB released from NLCs irradiated with 25 kGy of gamma radiation, exhibits a very similar effect. In contrast, the addition of PDGF-BB from non-irradiated nanoparticles and those irradiated with beta radiation (at both doses) results in significantly greater proliferation. The images acquired provide a preliminary understanding of wound closure, but a comprehensive analysis is required. The wound images were subsequently analyzed using Gen5 3.10 software, with the results presented in Fig. 10.

The baseline condition, in which no external stimulus was applied, reached 60% confluence. In contrast, the addition of PDGF-BB from non-irradiated NLCs resulted in nearly 100% confluence in the wound area. When PDGF-BB from NLCs irradiated with beta radiation was used, the 12 kGy dose achieved confluence levels comparable to those of non-irradiated nanoparticles. However, at the 25 kGy dose, confluence slightly decreased to 90%. This can be attributed to the dose-dependent effect of beta radiation, where higher doses have a more pronounced impact on PDGF-BB [27]. Therefore, the use of 25 kGy beta radiation results in a slight reduction in the bioactivity of the encapsulated factor.

Conversely, when analyzing the effect of PDGF-BB from NLCs irradiated with 25 kGy of gamma radiation, a different behavior was observed. The confluence in the wound area reached 70%, slightly higher than the baseline condition. PDGF-BB maintained some of its biological activity after irradiation, but showed a significant reduction in efficacy. This decline may be attributed to the ability of gamma radiation to fragment proteins [85], leading to the loss of their structure and, consequently, their bioactivity. Furthermore, gamma radiation also induces the formation of crosslinks [86], exacerbating this detrimental effect. The combined actions of both mechanisms could explain the observed reduction when irradiated with gamma radiation.

These data suggest that beta radiation has a lesser impact on PDGF-BB encapsulated within the NLCs. Conversely, the exposure to gamma radiation causes a decrease in the bioactivity of the growth factor. This phenomenon may be due to the greater influence of gamma radiation on the physical, mechanical and thermal properties compared to beta radiation [86, 87]. Due to this greater impact, radiation may induce structural changes in part of the growth factor. Consequently, this would interfere with its binding to receptors, resulting in decreased activity and therefore reduced cell proliferation.

Finally, a table (Table 2) is presented, summarizing the key results obtained throughout the experiments conducted in this study. This table aims to provide an overview and concise summary of how both types of radiation affect the NLCs, facilitating quick interpretation and comparison.

**Table 2:** Summary table of the effects of 12 kGy and 25 kGy of beta radiation, and 25 kGy of gamma radiation on VEGF165-loaded and PDGF-BB-loaded NLCs

		Beta Radiation		Gamma Radiation
		12 kGy	25 kGy	25 kGy
VEGF165-loaded NLCs	Mean Diameter	No changes	No changes	Size reduction
	Zeta Potential	No changes	No changes	No changes
	Polydispersity Index	No changes	No changes	Increases the value
	Release Profile	No changes	10% decrease is detected	50% decrease is detected
	Bioactivity	Slight decrease in bioactivity is observed, with the most effective concentration shifting from 2.5 to 5 ng/mL	Slight decrease in bioactivity is observed, with the most effective concentration shifting from 2.5 to 5 ng/mL	Loss of bioactivity, resulting in the absence of an angiogenic effect
PDGF-BB-loaded NLCs	Mean Diameter	No changes	Size reduction	Size reduction
	Zeta Potential	No changes	Slight reduction in zeta potential	Slight reduction in zeta potential
	Polydispersity Index	No changes	No changes	Increases the value
	Release Profile	50% decrease is detected	One-third decrease is detected	Only a 3% is detected
	Bioactivity	The bioactivity of the factor remains unchanged	Slight decrease in bioactivity is observed, with confluence reduced to 90%	Decrease in bioactivity is observed, with confluence reduced to 70%

## Conclusions

Osteoarticular diseases affect millions of people worldwide and are characterized by a reduction in the metabolic activity of bone tissue, leading to a reduction in bone and joint function. The incorporation of growth factors has emerged as an innovative therapeutic strategy to enhance healing [88, 89]. In this study, sterile NLCs loaded with VEGF165 and PDGF-BB were developed to evaluate their potential in the treatment of osteoarticular diseases. The synthesis and characterization of the NLCs revealed that these particles possessed suitable physicochemical properties and exhibited no cytotoxicity at concentrations below 250 µg/mL. In addition, the NLCs showed promising stability, allowing storage at 4 ºC for a month without significant changes in their properties, suggesting their potential for large-scale production in the future.

Research on sterilization using beta and gamma radiation revealed that beta radiation at a dose of 12 kGy is the optimal method for sterilizing NLCs. This method preserves the physicochemical integrity of the nanoparticles and minimizes the decrease in factor release and bioactivity. Previous studies support that the use of low-dose beta radiation is effective for terminal sterilization, without compromising the properties of the nanoparticles [90]. In contrast, gamma irradiation, particularly at higher doses, had a negative impact on factor release and bioactivity, which makes it less suitable for this purpose. Therefore, in future studies it could be interesting to explore reducing the dose used in the case of beta radiation to determine whether it can effectively sterilize the batch while minimizing its impact on the bioactivity of the factors. On the other hand, for gamma radiation, an intermediate dose between 12 kGy and 25 kGy could be considered, as the 12 kGy dose was insufficient to sterilize the batch. The results indicate that NLCs sterilized with beta radiation maintained high biological activity, with peak activity observed at concentrations of 5 ng/mL for VEGF165 and 50 ng/mL for PDGF-BB. VEGF165 promotes angiogenesis, enhancing blood and nutrient supply, while PDGF-BB stimulates cell proliferation, facilitating regeneration of the affected tissue.

Furthermore, the potential of NLCs loaded with VEGF165 and PDGF-BB extends to emerging applications in tissue engineering and 3D bioprinting. Scaffolds that contain NLCs may provide an optimal environment for cell proliferation and the formation of new blood vessels, which are critical for tissue and organ repair in regenerative medicine. This combined technology may open new opportunities for tissue repair and regeneration in regenerative medicine, improving treatment efficacy and providing advanced solutions for tissue defects.

In conclusion, the combined use of sterile NLCs loaded with VEGF165 and PDGF-BB not only represents a significant advancement in the treatment of osteoarticular diseases, but also opens new possibilities for their application in tissue engineering and 3D bioprinting. Adjusting the concentrations of these growth factors and tailoring the treatment to the specific needs of each patient could produce a synergistic effect, substantially enhancing prognosis and accelerating the healing process.

## Electronic supplementary material

Below is the link to the electronic supplementary material.

Supplementary Material 1

## Data Availability

The datasets generated and analyzed during the current study are available from the corresponding author upon reasonable request.
