# Supplementary material for: Effects of beta and gamma radiation sterilization on growth factor-loaded nanoparticles: an innovative approach for osteoarticular disorders treatment
Source: Drug Deliv Transl Res. 2025 Mar 11;15(10):3716–36. doi: 10.1007/s13346-025-01829-5 (PMC12397139; doi:10.1007/s13346-025-01829-5)
Supplement: Supplementary file 1 — Supplementary Material 1 [file 13346_2025_1829_MOESM1_ESM.docx]

Supporting Information

**Effects of Beta and Gamma Radiation Sterilization on Growth Factor-Loaded Nanoparticles: An Innovative Approach for Osteoarticular Disorders Treatment**

*Jorge Ordoyo-Pascual^1,2,3^, Sandra Ruiz-Alonso^1,2,3^, Idoia Gallego^1,2,3^, Laura Saenz-del-Burgo^1,2,3,*^ and Jose Luis Pedraz^1,2,3,*^*

**Supplementary Material 1. Nano Differential Scanning Calorimetry**

Nano Differential Scanning Calorimetry (nanoDSC) was used to investigate the behavior of growth factors with an increase in temperature, with the aim of determining their denaturation temperatures. Initially, both the reference and sample cells of the NanoDSC (TA Instruments, New Castle, USA) were filled with the required volume of Phosphate Buffered Saline (PBS) (Gibco, San Diego, California, USA). Once charged, the nanoDSC was configured with the following parameters: a pressure of 3 atmospheres, a temperature range from 20 ºC to 120 ºC, and a temperature increase of 1 ºC per minute. Upon start-up, the equipment generated a straight line, which served as the reference baseline. Subsequently, the PBS was removed from the sample cell and the growth factor, either vascular endothelial growth factor 165 (VEGF165) or platelet-derived growth factor BB (PDGF-BB) dissolved in PBS, was added. Once the cell was filled, the instrument was configured in the same way and activated. The resulting graph showed temperature peaks, and by subtracting the reference baseline, the thermogram for each growth factor was obtained, as shown in Figure S1A. Within the illustration, can be discern two distinct graphs, where an increase in the heat rate signifies the occurrence of an endothermic process, while a decline in the heat rate corresponds to an exothermic process. It is noteworthy that the denaturation processes characterizing the observed growth factors manifest as endothermic reactions, thereby appearing as positive peaks on the graph. For VEGF165, a decrease in heat rate is observed at 100ºC, indicating an exothermic process. This is followed by a significant endothermic peak at 105 ºC, characteristic of the growth factor. Consequently, it can be concluded that the denaturation process takes place within the temperature range of 100 ºC to 105 ºC. In contrast, PDGF-BB exhibits a slight exothermic peak at 60 ºC, followed by an increase in heat flow rate culminating in a peak at 72 ºC. In this case, the broader peak suggests that the denaturation process initiates at 60 ºC and achieves its maximum heat rate at 72 ºC.

As the denaturation temperature of PDGF-BB coincides with the temperature required for lipid melting, an additional assay was performed. For this purpose, PDGF-BB dissolved in PBS was heated in a water bath at 85°C for 5 minutes. This temperature was selected because it corresponds to the maximum endothermic transition temperature of the growth factor, while the duration of 5 minutes was chosen based on the typical time required for complete lipid melting. After this period, the sample was analyzed using the same method as previously. The results are presented in Figure S1B, which shows that heating PDGF-BB to 85°C and subsequent analysis results in the loss of its characteristic peak. This is probably due to denaturation, confirming the sensitivity of the growth factor to these temperatures.


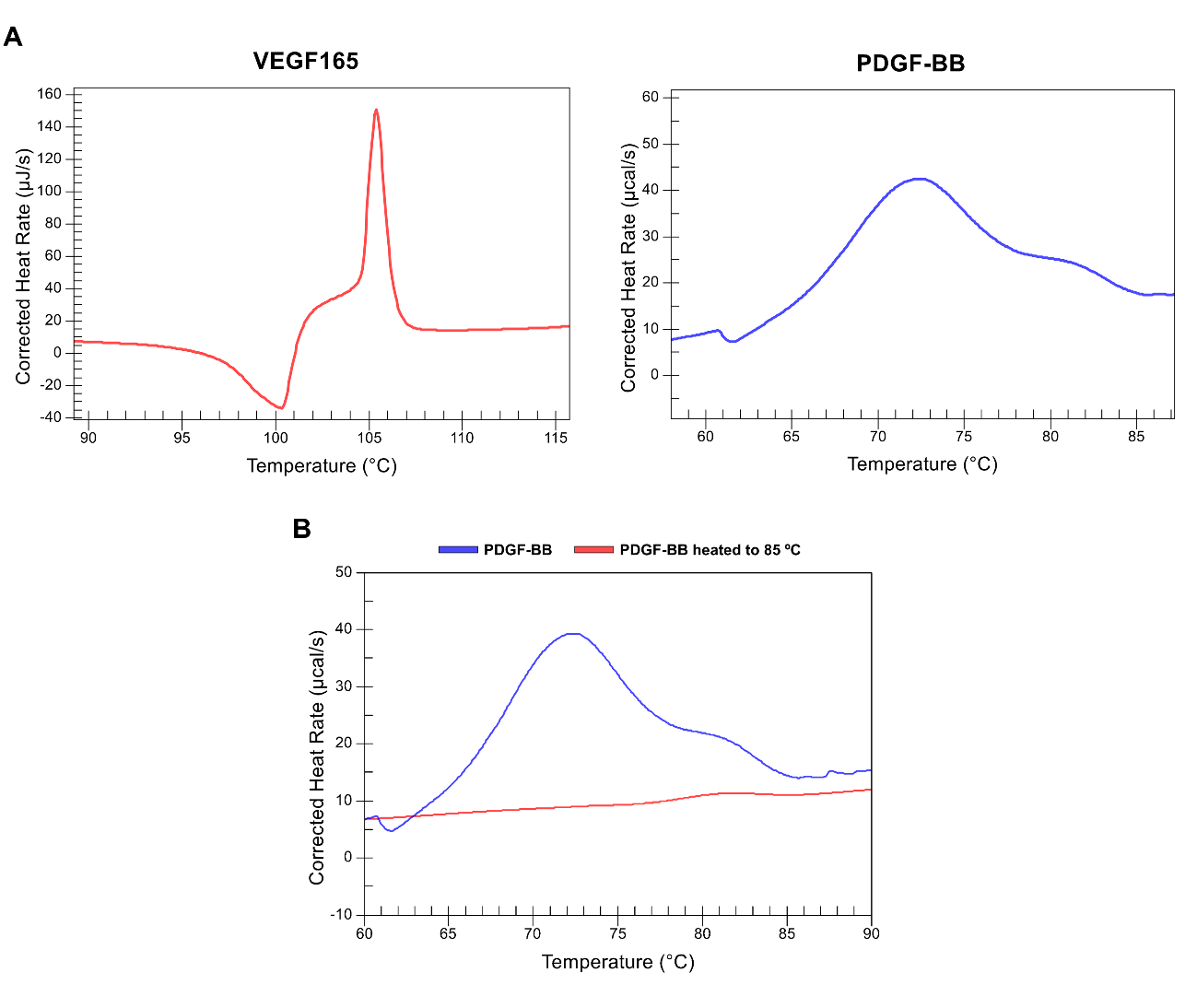


**Fig. S1** Nano Differential Scanning Calorimetry (NanoDSC) thermogram of **A)** VEGF165 and PDGF-BB and **B)** PDGF-BB heated to 85 °C for 5 minutes, compared with unheated PDGF-BB.

**Supplementary material 2. Angiogenesis assay**

VEGF165 is a molecule that exhibits biological activity at very low concentrations, making it important to determine the optimal concentration for maximum effect. For this purpose, an angiogenesis assay was performed on Human Umbilical Vein Endothelial Cells (HUVEC) (ATCC, Manassas, USA) as described in section 2.10.1 of the Materials and Methods. Different concentrations of VEGF165 (1 ng/mL, 2.5 ng/mL, 5 ng/mL and 10 ng/mL) obtained from the supernatants of nanostructured lipid carriers (NLCs) were tested to evaluate their respective effects. The images are summarized in Figure S2, and were analyzed to identify five parameters associated with angiogenesis. The comprehensive analysis of these results is presented in Figure S3.


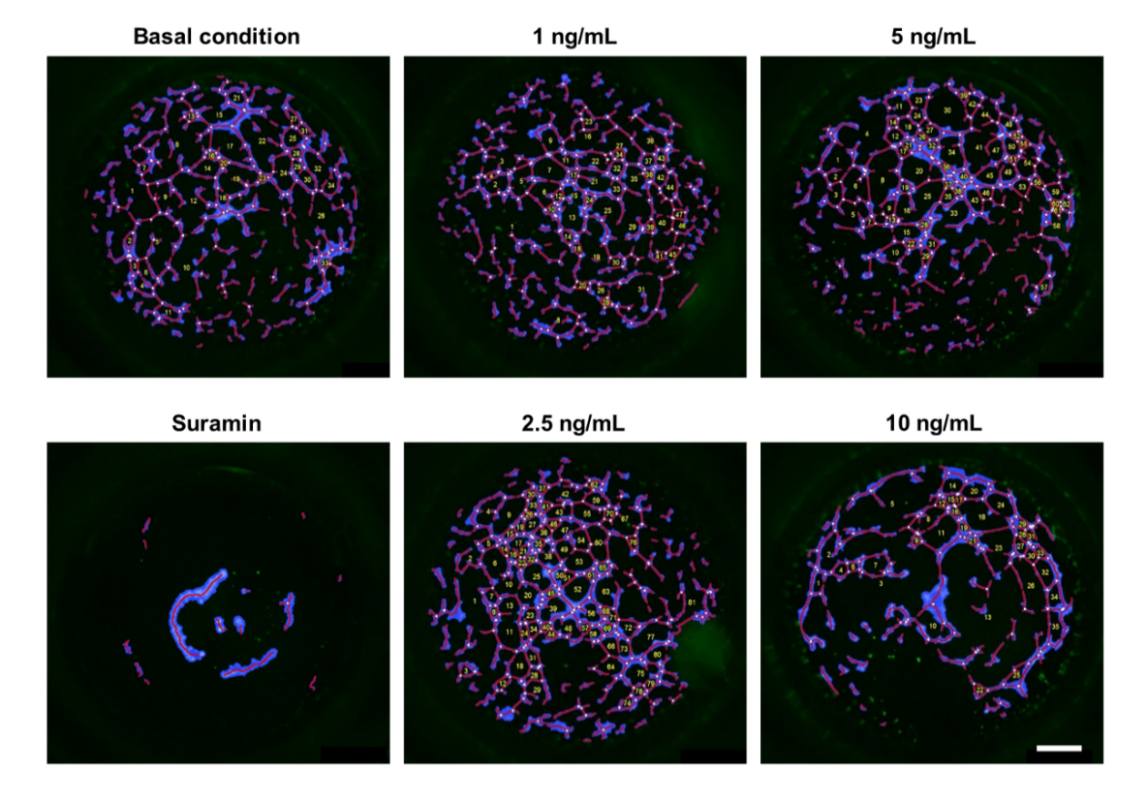


**Fig. S2** Analyzed images of the angiogenesis assay. Showing: in blue the covered area, the tubes in red, the branches as white dots and the vessels formed with a yellow number. Basal condition, suramin (negative control), 1 ng/mL, 2.5 ng/mL, 5 ng/mL and 10 ng/mL of VEGF165. Scale bar: 1000μm


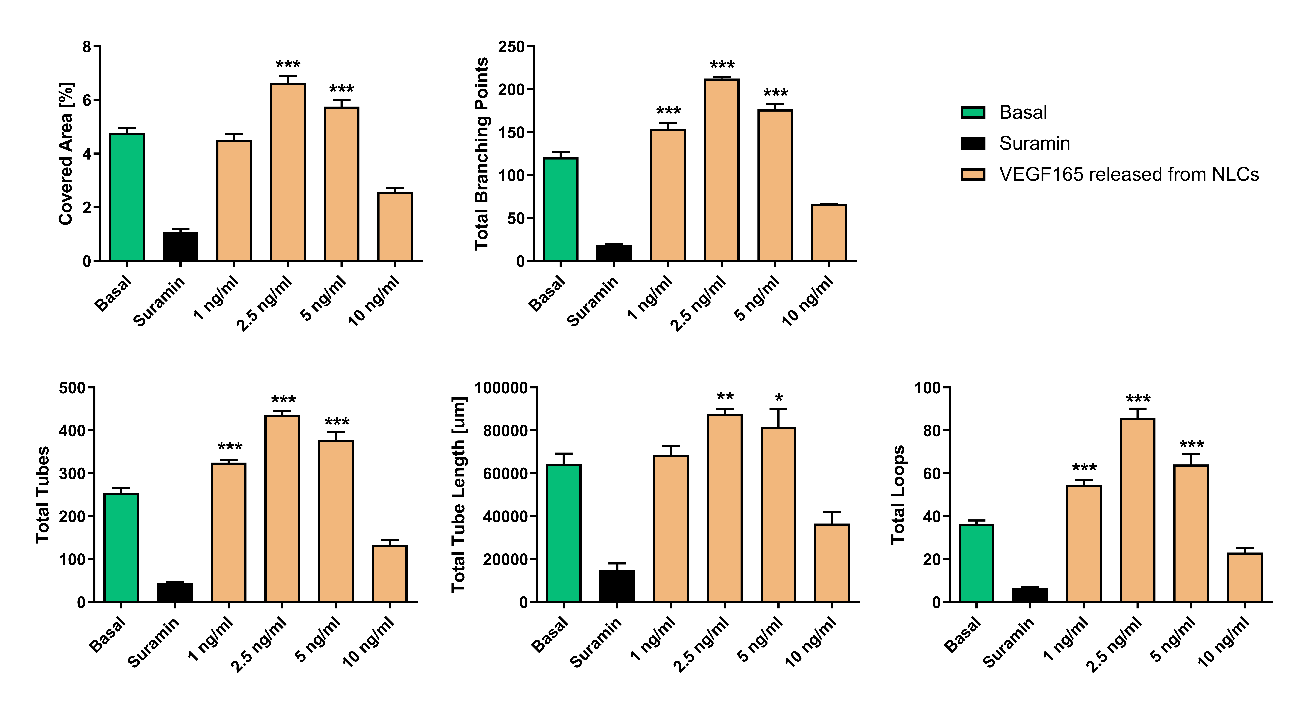


**Fig. S3** Quantitative parameters of the angiogenesis assay. Comparison of the effect of different concentrations of VEGF165 released from NLCs on HUVEC cells. * p < 0.05, ** p < 0.01 and *** p < 0.001 vs. basal condition. n=3

At a VEGF165 concentration of 1 ng/mL, significant differences in total branching points, total tubes, and total loops were observed. However, neither the covered area nor the total tube length show variations compared to the baseline condition. This suggests the onset of angiogenic effects at this concentration, indicating the initial stages of the process.

In contrast, both the 2.5 ng/mL and 5 ng/mL concentrations show significant differences across all parameters. These concentrations effectively promote tissue vascularization, with the peak effect observed at 2.5 ng/mL. Above this concentration, the effectiveness decreases as the concentration increases. Notably, at 10 ng/mL, the effect of VEGF165 was completely lost.

**Supplementary material 3. Wound healing assay**

PDGF-BB is a growth factor that plays a key role in the regulation of cell proliferation and migration. In order to determine the concentration at which maximum efficacy is achieved, a wound healing assay was performed on Human adipose tissue-derived stem cells (ADSCs) (provided by Viscofan). The assay followed the procedures described in section 2.10.2 of the Materials and Methods, employing different concentrations of PDGF-BB (10 ng/mL, 50 ng/mL and 100 ng/mL) obtained from the supernatant of the NLCs. Images showing wound closure progression are shown in Figure S4A. Subsequently, these frames were analyzed to generate a graph illustrating the percentage of wound closure, as shown in Figure S4B.


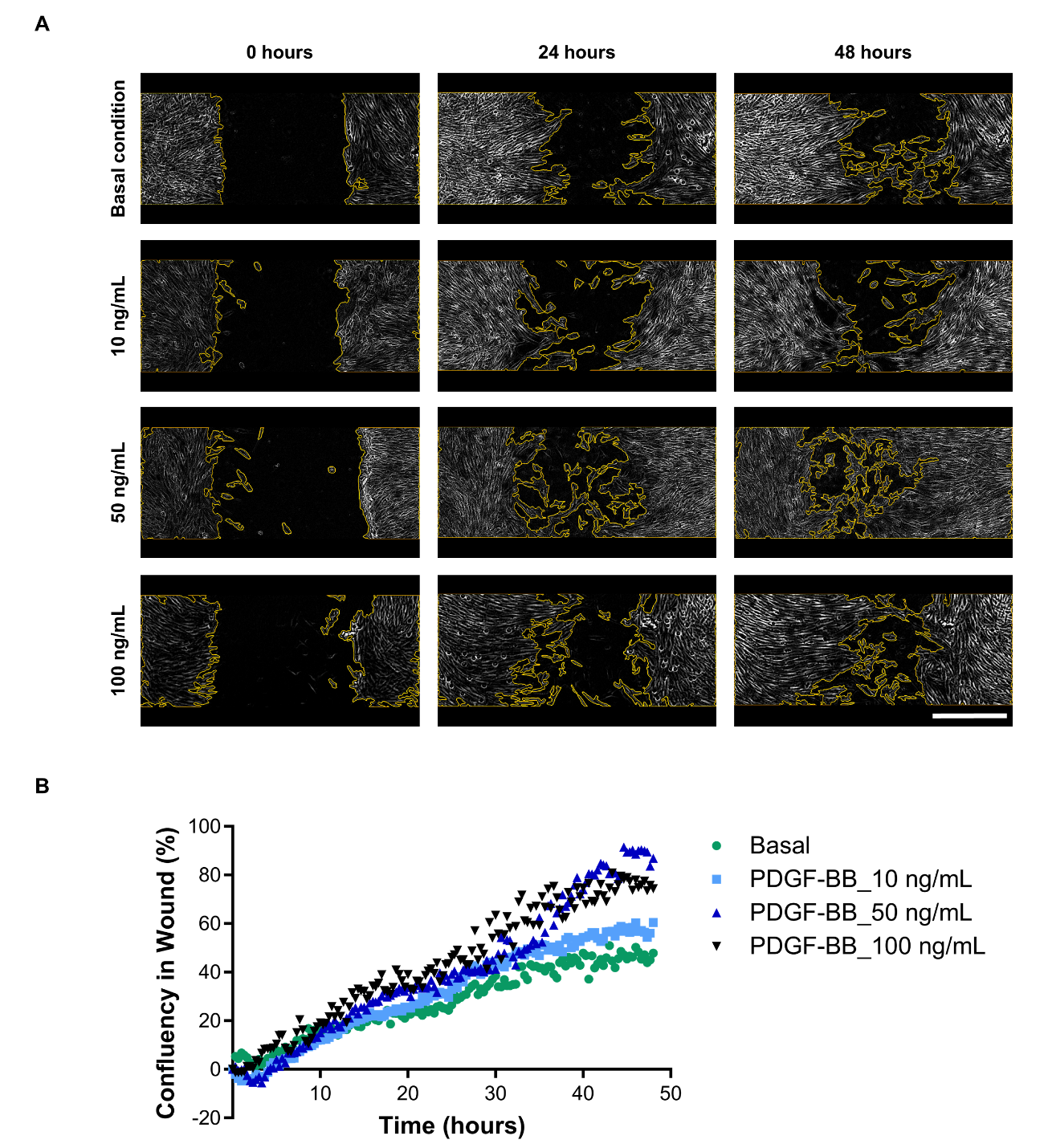


**Fig. S4** Wound healing assay in human ADSCs employing different concentrations of PDGF-BB released from NLCs. The released PDGF-BB concentration used were 0 ng/ml (basal), 10 ng/ml, 50 ng/ml and 100 ng/ml. **A)** Images of wound closure at time points 0, 24 and 48 hours. The yellow line delimits the area of cells detected in the image. Scale bar 1000 μm. **B)** Quantitative analysis of the confluence of human ADSCs in the wound. n = 3

The graph indicates that cell confluence in the wound area was approximately 40% when only culture medium was added. In contrast, under conditions where different concentrations of PDGF-BB from the NLCs were added, cell confluence consistently surpassed this level. Consequently, all concentrations of released PDGF-BB demonstrated biological activity. At a PDGF-BB concentration of 10 ng/mL, cell confluence increased slightly to 50%. However, at a concentration of 50 ng/mL, cell confluence achieved 90% wound closure. Conversely, at a higher concentration of 100 ng/mL, bioactivity decreased minimally, resulting in 80% cell confluence. This suggests that the peak activity for PDGF-BB released from NLCs is at 50 ng/mL. Any deviation from this concentration, either by decreasing or by increasing it, results in a reduction in bioactivity.
